# Sociodemographic Influences on Perceived Stress during Pregnancy: Results from the CCREOH Environmental Epidemiologic Study, Suriname

**DOI:** 10.3390/women2020014

**Published:** 2022-05-20

**Authors:** Aloysius Ph. Koendjbiharie, Ashna D. Hindori-Mohangoo, Wilco C. W. R. Zijlmans, Arti Shankar, Firoz Z. Abdoel Wahid, Hannah H. Covert, Maureen Y. Lichtveld, Stacy S. Drury

**Affiliations:** 1 Community Health Department, Regional Health Services, Paramaribo, Suriname; 2 Faculty of Medical Science, Anton De Kom University, Paramaribo, Suriname; 3 Foundation for Perinatal Interventions and Research in Suriname (Perisur), Paramaribo, Suriname; 4 Department of Environmental Health Sciences, School of Public Health and Tropical Medicine, Tulane University, New Orleans, LA 70112, USA; 5 Department of Biostatistics and Data Science, School of Public Health and Tropical Medicine, Tulane University New Orleans, LA 70112, USA; 6 Department of Environmental and Occupational Health, School of Public Health, University of Pittsburgh, Pittsburgh, PA 15261, USA; 7 Department of Psychiatry, School of Medicine, Tulane University, New Orleans, LA 70112, USA

**Keywords:** suriname, psychosocial stress, perceived stress, Cohen’s Perceived Stress Scale, pregnant women, sociodemographic factors

## Abstract

Screening for prenatal stress is not routine in Suriname, despite its significant impact on maternal and newborn health. This study assessed the prevalence of high perceived prenatal stress and its sociodemographic predictors in three geographic areas in Suriname. In this cross-sectional study, data from 1190 participants of the Caribbean Consortium for Research in Environmental and Occupational Health cohort study were analyzed. Cohen’s Perceived Stress Scale was completed during pregnancy to ascertain high perceived stress (cut-off score 20). The association between maternal sociodemographic factors and high perceived stress was examined using the chi-square test and logistic regression models; 27.5% of all participants had high perceived stress with statistically significant lower rates in Nickerie (18.8%) compared with Paramaribo (29.8%; *p* = 0.001) and the Interior (28.6%; *p* = 0.019). Maternal sociodemographic factors moderated the difference between the Interior and Nickerie. Participants from Paramaribo had statistically significant higher odds of high perceived stress compared to those from Nickerie, independent of their age and educational level (adjusted OR = 1.94; 95% confidence interval 1.32–2.86). Perceived stress during pregnancy is predicted by sociodemographic factors. These findings identified target groups for interventions in Suriname. Policy makers should consider integrating perceived stress assessment as a routine part of prenatal care.

## 1. Introduction

Evidence indicates that maternal prenatal psychosocial stress is associated with increased risk of adverse health outcomes in mothers and offspring, including adverse birth outcomes and poor social and neurodevelopment [1–3]. The fetal brain is especially vulnerable to maternal stress, due in part to the rapid neurodevelopment occurring in utero [4]. High maternal psychosocial stress is transmitted through both biological and behavioral factors to the developing fetus, influencing the developing fetal tissues and biological systems and ultimately contributing to adverse health outcomes [4].

Maternal prenatal psychosocial stress has been characterized by a spectrum of measures and exposures, including perceived stress, socioeconomic stress, anxiety, and depression [5,6] The most commonly considered factors in relation to maternal psychosocial stress are tied to exposure to daily tensions and worries, or sociodemographic stressors. Other factors related to psychosocial stress during pregnancy include exposure to traumatic events, abuse, domestic violence, and racism [5,6]. Low socioeconomic status (SES) and neighborhood stressors, including living in an area of high neighborhood disorder, high rates of crime and violence, and/or decreased access to needed resources or health care have also been shown to contribute to elevated maternal prenatal psychosocial stress [5,6]. In low- and middle-income countries (LMICs), studies have found prenatal maternal psychosocial stress rates in the 6–53% range [7,8]. Our study is expected to be the first to examine sociodemographic predictors of maternal psychosocial stress across three distinct geographic and cultural groups in Suriname. We focused on perceived stress as an indicator of psychosocial stress, which we defined as the emotional response of pregnant women to negative life events experienced in daily life.

Exposure of pregnant women to stressors for a short period of time is defined as acute prenatal perceived stress, while continuous or repetitive exposure to stressors may result in chronic perceived stress, which can impact the health of the pregnant women depending on her coping abilities and resources [9]. Maternal prenatal perceived stress is influenced not only by specific negative events, “stressors”, but also by the sociodemographic environment in which the stressor occurs [10]. Pregnant women with different cultural backgrounds may have different perceptions, as well as expressions, of perceived stress. These sociodemographic differences likely contribute to the observed variation in prevalence across cultures. Studies have reported the prevalence of prenatal stress in study participants as 6% in the United States [11], 11.6% in Ethiopia [12], 28.6% in Ghana [13], 33% in India and in Saudi Arabia [14,15], and 34% in Nepal [8]. Sociodemographic factors such as marital status, income, education, perceived social status, age, household size, and ethnic background have been hypothesized to influence the risk of high maternal prenatal stress [10,16]. These variables, individually or in combination, may either buffer or further contribute to maternal stress, thereby exceeding the coping capacity in pregnant women and resulting in differences in maternal perceived stress by cultural background. As a first step in enhancing maternal-child health, a clearer understanding of the association between sociodemographic factors and maternal prenatal perceived stress among different ethnic groups in a multi-cultural country such as Suriname is needed. Given the wide variability in the prevalence of maternal prenatal psychosocial stress across cultures, examining the prevalence by both culture and geographic area, as well as the correlates with sociodemographic variables, is expected to guide future efforts seeking to diminish the cross generational impact of maternal prenatal stress. To date, there is a paucity of information about the social, cultural, and demographic factors that may elevate a pregnant woman’s risk in developing countries such as Suriname.

Suriname is located in the northern part of South America, between the Atlantic Ocean in the north, Brazil in the south, French Guyana in the east, and Guyana in the west. The multi-ethnic population of 541,638 consists of 27.4% Hindustani (with origins in the Indian subcontinent), 21.7% Tribal people (formerly Maroons), 15.7% Creole, 13.7% Javanese, 13.4% Mixed, and 3.8% Indigenous people (Amerindians) [17]. Tribal people and Creoles are descendants of Africans. Creoles live primarily in Paramaribo, the capital, whereas Tribal people initially set up communities in the interior tropical rainforests but due to socioeconomic circumstances have migrated to areas in Paramaribo and other districts in the coastal area. The remainder represents Chinese and Caucasians [17]. The majority of the Surinamese population lives in the urban districts of Paramaribo and Wanica. One fifth of the total population lives in the rural areas (Nickerie, Coronie, Saramacca, Commewijne, and Para). Fourteen percent of the population resides in the southern eighty percent of the country, which primarily comprises the Amazonian rainforest and is referred to as the Interior [17].

Suriname can be divided into three distinct areas, based on geographic, socio-economic, and cultural characteristics: the interior; the rural coastal area, such as the district of Nickerie; and the urban coastal area, such as the district of Paramaribo. Tribal and Indigenous peoples who live in villages along the rivers inhabit the Interior region. Their primary means of existence includes small-scale agriculture, goldmining, hunting, fishing, and logging. In Nickerie, located in the northwest of the country, the primary economic focus is larger-scale agriculture, particularly rice, as this region is the largest rice producer in Suriname. In Nickerie, the largest ethnic group is the Hindustani (60%), followed by the Javanese (17%) [17]. The majority of the Surinamese population resides in the capital, Paramaribo, where the primary economic focus is trade and small industries, and companies engage in food production and processing, gold processing, and producing other products for the domestic market. The largest ethnic groups in Paramaribo are Creole (26%) and Hindustani (23%) [17]. The different economic structures and significant differences in racial and ethnic populations across these three geographic areas provide an important context in which to evaluate the sociodemographic correlates of maternal prenatal stress.

Despite the well-known health impacts of prenatal stress on mothers and children, prenatal screening for perceived stress is not part of routine prenatal care in Suriname. To date, national efforts have been focused on the prevention of pregnancy-related mortality. In Suriname, prenatal care is provided by family physicians, and midwives associated with a primary health care clinic. These health care providers currently lack a standardized approach to assess prenatal psychosocial stress. In addition, data on possible risk factors such as maternal sociodemographic factors are limited. Since psychosocial risk factors may be potentially modifiable, from a public health perspective, the identification of pregnant women who suffer from perceived stress is critical to both the development and implementation of intervention and treatment programs to improve mother and child health (MCH) in Suriname.

To address this gap in knowledge, our study aimed to (1) assess the prevalence of perceived stress among pregnant women from different cultural/ethnic groups living in three different geographic areas in Suriname, (2) describe the maternal sociodemographic determinants of high perceived stress in order to identify high-risk pregnant women and geographic areas, and (3) inform and thereby promote the integration of routine maternal prenatal perceived stress screening within the primary care setting. We hypothesized that maternal sociodemographic factors would predict perceived stress during pregnancy and that the prevalence of maternal perceived stress would differ across geographic areas in Suriname.

## 2. Materials and Methods

### 2.1. Study Design

The Caribbean Consortium for Research in Environmental and Occupational Health (CCREOH) study is a prospective environmental epidemiologic cohort study in Suriname that addresses the impact of chemical and non-chemical environmental exposures in mother/child dyads [18]. In the cross-sectional study reported on here, we focused on perceived stress as a non-chemical environmental exposure during pregnancy. Pregnant women were recruited during the first or early second trimester of pregnancy from three geographic areas of Suriname: Paramaribo, Nickerie, and the Interior. From December 2016 to July 2019, eligible pregnant women were recruited from hospitals, prenatal clinics, and midwife facilities of the Regional Health Services, and in the Interior at multiple health care clinics of the Medical Mission Primary Health Care Suriname. Women were eligible if they were 16 years or older; spoke Dutch, Sranan Tongo, Saramaccan, or Trio; had a singleton gestation; planned to give birth at one of the study sites; and provided written informed consent.

### 2.2. Study Population

In total, 1200 women were recruited; 10 were excluded because they did not meet the inclusion criteria. Analyses for this study were based on the total cohort of 1190 eligible pregnant women, who completed their first prenatal assessment for the CCREOH study during the first or second trimester of pregnancy. Questionnaires were administered by trained recruiters through face-to-face interviews with participants using encrypted iPads. As part of this first assessment, data on maternal sociodemographics, perceived social status, and perceived stress were collected.

### 2.3. Outcome (High Perceived Stress)

Perceived stress is defined as self-reports of the presence of daily stress during pregnancy based on Cohen’s Perceived Stress Scale (CPSS). This 10-item questionnaire asks about the feelings and thoughts experienced during the last month in situations defined as uncontrollable and unpredictable. The CPSS is a reliable instrument to measure perceived stress in men and women (including pregnant women) and was validated in multi-racial and multi-ethnic populations [19]. Solivan et al. found a positive correlation in their study between the CPSS and another instrument used to measure perceived stress [20]. Additionally, the CPSS was positively correlated with the Edinburgh Depression Scale. The Cronbach’s alpha for the CPSS is between 0.84 and 0.86 [21]. The 75th percentile was used as a cut-off level to characterize participants as ‘high perceived stress’ (score 20–40) and as ‘low perceived stress’ (score 0–19).

### 2.4. Recruitment Location

Since eligible women were recruited from three different geographic areas of Suriname, these regions were explored as the primary predictor and defined as Paramaribo vs. Interior vs. Nickerie. Some participants living in the geographic areas bordering Paramaribo (districts Commewijne, Para, Saramacca, and Wanica) were analyzed as participants from Paramaribo; participants from district Coronie (bordering Nickerie) were analyzed as participants from Nickerie; and the Interior included participants from districts Sipaliwini, Brokopondo, and Marowijne.

### 2.5. Maternal Demographics

Ethnic background, age, parity, educational level, household income, household size, marital status, and perceived social status were explored as covariates. Ethnic background, based on self-report of how the participant identified herself, was first categorized based on the main ethnic groups in Suriname (Creole, Hindustani, Indigenous, Javanese, Tribal people, and Mixed). Age at intake was explored as both continuous and categorical (16–19 vs. 20–24 vs. 25–29 vs. 30–34 vs. 35+ years). Parity was defined based on the number of previous live births and dichotomized into 0–3 vs. 4+ previous live births. The highest completed educational level was based on the woman’s report of the highest grade and degree completed and categorized as not educated/primary education vs. lower secondary/vocational vs. upper secondary/vocational vs. tertiary. Household size, including the participant, was studied as a dichotomous variable (<3 vs. 3+ persons in household). Information on monthly household income in Surinamese Dollars (SRD) was first analyzed in four subgroups (<800 vs. 800–1499 vs. 1500–2999 vs. 3000+ SRD). Marital status was dichotomized as married or living with partner vs. unmarried or not living with a partner. Based on the association with the outcome of high perceived stress, and due to small sample size for some subgroups of covariates, the following dichotomous subgroups were used for bivariate and multivariate analyses: ethnic background (Creole and Tribal ethnicity (Black women) vs. all other and mixed ethnicities); age at intake (16–19 vs. 20+ years); educational level (less educated (not educated or primary or lower secondary/vocational level of education) vs. more educated (upper secondary/vocational or tertiary); and household income (< 3000 vs. 3000+ SRD (USD 210)). The participants’ perceived social status during the face-to-face-interview was also explored as a covariate. The MacArthur Scale of Subjective Social Status was used to measure perceived social status using a scale ranging from 0–100. The scale measures the perception that individuals have of their place in the society in relation to others. The participant was asked to move a block along a line numbered from 0 to 100 to indicate where she thinks she is at this moment in her life, relative to other people in her community. It was analyzed as a continuous variable and subsequently dichotomized based on the mean minus 1 standard deviation to define low (0–30) vs. normal perceived social status (31–100).

### 2.6. Statistical Analyses

Descriptive statistics of the study population were calculated and stratified for the geographic areas (Paramaribo, Interior, and Nickerie) and presented as means with standard deviations for normally distributed continuous variables or medians with interquartile range for skewed distributed continuous variables and as proportions for categorical variables. Crude associations between maternal sociodemographic variables and high perceived stress were explored using the chi-square test; differences in proportions were tested with the two-sample test. Risk ratios (RR) with 95% confidence intervals (95% CI) and *p*-values were calculated for high perceived stress. Logistic regression models were computed to explore whether differences in high perceived stress between the geographic areas could be explained by the participants’ ethnic background or other maternal sociodemographic variables. In these models, high perceived stress was analyzed as a dichotomous outcome (yes vs. no) and geographic area as the primary predictor (Paramaribo vs. Interior vs. Nickerie). In subsequent models, the association between geographic areas and high perceived stress was adjusted stepwise for ethnic background, age/parity, educational level/household income/household size/marital status, and perceived social status. The results were expressed as odds ratios (ORs) with 95% CI. *p*-values < 0.05 were considered to be statistically significant. Because 16% of participants had missing MacArthur values, the distribution of maternal sociodemographic variables and perceived stress was compared between participants with and without MacArthur scores (Supplementary table S1). All analyses were completed using the Statistical Package for Social Sciences (SPSS), version 27.0 for Windows (IBM Corp. Released 2020, Armonk, NY, USA).

## 3. Results

The general characteristics of the study population, stratified for the geographic areas, are presented in Table 1. Of all 1190 participants, 62% were recruited in Paramaribo (*n* = 738). The remaining participants were recruited in the Interior (19.2%; *n* = 228) and in Nickerie (18.8%; *n* = 224).

### 3.1. Demographics

The three geographic areas differed in terms of statistical significance for all maternal socio-demographics. For the Interior, higher proportions were observed of participants aged 16–19 (22.4% vs. 9.9%/12.1% in Paramaribo/Nickerie), participants with high parity > 3 (28.0% vs. 9.6%/6.3% in Paramaribo/Nickerie), less educated participants (74.9% vs. 11.8%/9.0% in Paramaribo/Nickerie), household size of at least 3 persons (95.6% vs. 87.1%/86.9% in Paramaribo/Nickerie), participants with a household income less than 800 SRD (53.2% vs. 6.6%/7.3% in Paramaribo/Nickerie), and participants with low perceived social status (45.2% vs. 9.9%/ 5.2% in Paramaribo/Nickerie). In Nickerie, there was a higher proportion of mothers between the ages of 20 and 24 years (30.4% vs. 21.1%/21.5% in Paramaribo/Interior), participants with low parity (93.7% vs. 90.4%/72.0% in Paramaribo/Interior), and households of less than 3 persons (13.1% vs. 12.9%/4.4% in Paramaribo/Interior). However, in Paramaribo there was a higher proportion of tertiary educated participants compared to Nickerie and the Interior (20.3% vs. 11.8%/0.4% in Nickerie/Interior). The distribution of ethnic groups also differed significantly between the three geographic areas, with almost all participants in the Interior having an Indigenous or Tribal ethnic background (93%), 46.6% of participants in Nickerie having a Hindustani ethnic background, and 32.2% of participants in Paramaribo having a Creole ethnic background.

### 3.2. Predictors of High Perceived Stress

Table 2 shows the differences by high and low perceived stress for each maternal sociodemographic characteristic. The percentage of participants with high perceived stress differed statistically significant by geographic area (*p* = 0.006). Participants from Paramaribo (29.8%) and from the Interior (28.6%) were more likely to report high perceived stress compared to participants from Nickerie (18.8%): RR (95% CI), respectively, 1.58 (1.18–2.13) and 1.52 (1.08–2.14). The observed rates of high perceived stress between participants from Paramaribo and those from the Interior were not significantly different: RR = 1.04; 95% CI (0.82–1.32).

Other statistically significant predictors of high perceived stress were participants with Creole and Tribal ethnic backgrounds (RR = 1.31; 95% CI 1.09–1.58; *p* = 0.004), teenage participants aged 16–19 years (RR = 1.46; 95% CI 1.15–1.84; *p* = 0.003), participants with high parity (RR = 1.35; 95% CI 1.06–1.72; *p* = 0.021), less educated participants (RR = 1.58; 95% CI 1.29–1.94; *p* < 0.001), participants with lower household income (RR = 1.36; 95% CI 1.09–1.70; *p* = 0.006), and participants with low perceived social status (RR = 1.33; 95% CI 1.04–1.70; *p* = 0.030). Household size (*p* = 0.453) and marital status (*p* = 0.062) were not significant predictors of high perceived stress.

Table 3 presents crude and adjusted logistic regression models for high perceived stress. Participants from Paramaribo and the Interior had significantly increased odds of high perceived stress than those from Nickerie (crude OR, respectively, 1.82 (95% CI 1.25–2.65) and 1.72 (1.10–2.68)). The observed higher odds for high perceived stress for participants from the Interior vs. those from Nickerie were explained by their age, parity, and ethnic background (adjusted model 2: OR = 1.41; 95% CI (0.87–2.27)). After controlling for other covariates (adjusted model 4), the differences between Paramaribo and Nickerie remained statistically significant with participants from Paramaribo, having higher odds of high perceived stress (adjusted OR = 1.68; 95% CI 1.08–2.62). The final multivariate model revealed that participants from Paramaribo, teenage participants, and less educated participants had independent higher risks of high perceived stress ORs, respectively 1.94 (95% CI 1.32–2.86), 1.57 (95% CI 1.08–2.28), 1.90 (95% CI 1.41–2.55). Relevant interactions between sociodemographic factors were explored, and no significant interaction was found.

## 4. Discussion

To our knowledge, this is the first study to explore the sociodemographic predictors of maternal prenatal perceived stress across ethnic groups and geography in Suriname. Our results provide novel evidence that sociodemographic factors contribute to the regional differences in maternal prenatal perceived stress in Suriname. We found that across the three geographic areas in Suriname, approximately 1 out of 4 pregnant women self-reported high perceived stress. The rates of high perceived stress exhibited statistically significant regional differences ranging from a low of 18.8% in Nickerie to a high of 29.8% in Paramaribo.

The high percentage of participants reporting high stress levels, and the statistically significant differences by geographic area, are both consistent with previous studies in LMICs which reported rates of psychosocial stress ranged from 6% to 53% [7,8]. Worldwide, about 10% of pregnant women in high-income countries experience some form of perinatal mental disorder, while in LMICs the prevalence is higher, with an average of 15.6% [9]. Comparisons to other studies, however, should be made carefully. For example, our study focused on prenatal maternal perceived stress, while Fisher et al. included other forms of perinatal mental disorders, such as anxiety and depression [9]. The average stress rate in our cohort was 27.5%, while Woods [11] reported 6% in a US cohort. Thus, almost 3 in 10 pregnant women had high perceived stress in the Suriname cohort, while less than 1 in 10 did in the US cohort. In LMICs, pregnant women are exposed to more psychosocial stressors compared to developed countries. Inequities in predictors of health and the social, cultural, and political contexts of pregnant women in LMICs may negatively influence women’s mental health. [9,22]. Moreover, the variability in the prevalence of maternal prenatal psychosocial stress reported across studies may reflect the broad characterization of maternal prenatal psychosocial stress, the study design, the instrument employed to measure psychosocial stress, and the specific type of disorder measured (i.e., perceived stress, anxiety, and depression). Inadequate consideration of sociodemographic differences for surveys, or differences in experiences and exposures of psychosocial stress by geographic area and cultural group, may also reflect these reported differences in the prevalence of maternal prenatal stress [8].

High prenatal perceived stress has a statistically significant association in the bivariate analyses with geographic area, ethnic background, age, parity, education income, and perceived social status. Younger age, lower income, lower educational level, and lower socio-economic status were reported in other studies as maternal characteristics associated with psychosocial stress [8,9,11,23]. Our results concur with these studies.

Results in the bivariate analyses suggest that pregnant women living in Paramaribo, followed by pregnant women in the Interior, had higher risk of perceived stress compared to pregnant women in Nickerie. However, after adjustment for key sociodemographic variables in the multivariate logistic regression analyses, while statistically significant differences in prenatal stress remained between Nickerie and Paramaribo, statistically significant differences were no longer present between Nickerie and Interior. These results suggest that sociodemographic factors, particularly maternal age and parity, are key predictors for prenatal stress in the Interior.

We observed the highest prevalence for high stress in the youngest age group (16–19 year) in our study. A higher proportion of teen pregnancies were reported in the Interior (22.4% vs. 12.1% in Nickerie). Our findings are also in agreement with previous studies that described a higher prevalence of psychological stress among pregnant teenagers [23,24]. The resources and capacity to cope with the stress are generally lower in younger pregnant women compared to older pregnant women [25]. Intimate partner issues may contribute to high perceived stress in pregnant teenagers, such as lack of partner and having a young partner who rejects paternity or is unsupportive and uninvolved [25]. Partner support may play an important role in the psychological well-being of pregnant teenagers [26]. Other contributing factors to high perceived stress in teenagers may be experiencing feelings of shame, stigmatization, loneliness, and helplessness [25].

Parity was a key determinant for prenatal stress in participants in the Interior, in our study. Participants in the Interior had five times more children (4+) than participants in Nickerie according to our results. Our findings are in line with a previous study in Ethiopia by Engidaw et al. [12]. Raising many children can be a source of stress due to financial constraints and lower social economic status, especially for single mothers. Mothers with a poor obstetric history may be worried and have tensions towards the current pregnancy [12].

Previous studies have reported education as a predictor of maternal prenatal stress [24,27]. Education can improve coping abilities in pregnant women by providing resources such as knowledge and skills to deal with psychosocial stressors. In our study, less educated women had an overall increased risk for high perceived stress compared with more educated women (RR = 1.58; 95% CI 1.29–1.94). Educational level was one of the independent predictors of high perceived stress in pregnant women.

According to our analyses, in adjusted model 1, the differences in stress between participants in the Interior and Nickerie and between participants in Paramaribo and Nickerie can be explained by the ethnic composition of these three areas. However, when adjusting for maternal age and parity in adjusted model 2, ethnic background was not a significant predictor anymore and the significance between the geographic areas, the Interior, and Nickerie was lost, although participants in the Interior continued to have higher odds of stress. We suggest that this was due to a joint effect of these three sociodemographic variables.

In the multivariate logistic regression analyses, the differences in prevalence in the three areas in Suriname were partially explained by the sociodemographic composition in these areas. However, even after accounting for these covariates, Paramaribo continued to have statistically significant higher levels of maternal perceived stress than Nickerie. Participants from Paramaribo, teenage participants and less educated participants had independent higher risks of high perceived stress. These results suggest that, irrespective of the specific sociodemographic factors tested, mothers residing in Paramaribo are at higher risk for prenatal perceived stress and that other factors, not specifically measured in this study, might contribute to maternal stress in Paramaribo.

Stress perception is in general greater in women in urban areas compared to women in rural areas [28]. Attributable factors to the differences in maternal stress between Paramaribo and Nickerie could include differences in contextual characteristics in these two locations. Literature suggests that urban life could be a continuous source of stress due to urban factors such as high population density, increased concentration of motor traffic, and traffic congestion [29]. High population density, social density, and spatial density are associated with experiences of noise nuisance from neighbors and traffic; crime; and violence [29–31]. High population density may affect interpersonal relationships and mental health in the general population [29,31,32], including pregnant women in Paramaribo. Paramaribo is the smallest district in terms of land area, 182 km^2^, but has the largest population in Suriname (240,924). Compared to Nickerie, with a land area of 5,353 km^2^ and a population of 34,233, the population density of Paramaribo (1323.8/km^2^) is more than 200 times greater than Nickerie (6.4/km^2^) [34]. Reports of the Ministry of Justice and Police, Department of Crime Information, also indicate that crime rates in Paramaribo (116.7/1000) in 2018 were 4 times higher than in Nickerie (28.5/1000) [33].

In rural areas, such as Nickerie, higher levels of social support from the partner and family members may be available for the pregnant women compared to Paramaribo [9]. Social support can act as a positive moderator on how stress is perceived or experienced by pregnant women. A higher level of social support is associated with lower levels of stress, compared to no social support [34,35].

Future studies examining these factors are needed to better understand the observed differences in maternal perceived stress and are a critical next step in public health efforts directed at decreasing maternal stress and its subsequent effects on maternal and child health. These findings, when combined with the overall high rates of high perceived stress, support the implementation of universal screening for prenatal perceived stress in Paramaribo.

### 4.1. Limitations

There are several limitations to our study. Maternal sociodemographic data and perceived stress were obtained through self-report questionnaires. This approach may be associated with reporting biases due to socially desirable responses and personal privacy issues. This could also result in misclassification and the underestimation of the prevalence of stress in this group of women. Another challenge is the lack of cross-culturally valid perinatal perceived stress screening and diagnostic instruments in Suriname, particularly during the antepartum period. The CPSS has been translated into many other languages, so its use is not limited to English-speaking countries. Van Eck et al. translated the 10-item version of the CPSS into Dutch and showed that the norms for perceived stress were comparable to those of the U.S [36]. The broad use of the CPSS suggests it applicability in Suriname [37]; however, there is a clear need for researchers to refine and rigorously evaluate the predictive validity and reliability of this culturally sensitive tool in a multi-linguistic and multi-cultural country such as Suriname. In our study, the recruiter asked the questions in a local language if the participants did not fully understand the questions in Dutch. The CPSS measured perception of stressors in the past month. As such, we are not able to determine if higher perceived stress was related to maternal mental health problems prior to pregnancy or of longer duration. The data analyzed in our study were from one point in time, which limited our ability to examine the differential impact of stress across the course of pregnancy. This study did not obtain data on maternal lifetime history of mental health or family history of mental illness. Other factors that could influence perceived stress, such as chemical exposures, maternal coping ability, and resources, were not evaluated in this study. Some participants (16%) were missing MacArthur scale data. However, analyses performed with and without the MacArthur scale show that social status did not have an impact on the findings in this study; social status was not an independent predictor of high perceived stress in the multivariate analyses (Table 3). Data on births from each geographic study area were not available. We therefore could not weight the study sample as the proportion of births in each area.

### 4.2. Strengths

To our knowledge, this study is the first to report on the sociodemographic correlates of prenatal maternal perceived stress across three distinct geographic and cultural groups in Suriname. The large sample size (*n* = 1190), and the geographic, ethnic, and cultural diversity of the study population, combined with the wide range of sociodemographic factors, enhances generalizability. Our findings provide important information to inform and promote public health interventions in high-risk pregnant women, and teenagers of African descent and with lower education, and identify the need for universal screening in Paramaribo as well as future areas of research to improve maternal and child health.

## 5. Conclusions

We found significant regional differences in the rates of maternal prenatal perceived stress. Geographic area was the primary predictor of high maternal prenatal perceived stress among women in Suriname. For pregnant women in the Interior, sociodemographic factors could explain these regional differences. However, in Paramaribo these same factors failed to account for regional differences, suggesting that other factors in Paramaribo contribute to high maternal stress. If we consider that there are approximately 10,000 births each year in Suriname, based upon observed rates in this study, it is estimated that almost 2,700 pregnant women each year experience high perceived stress. Health professions education is needed to inform healthcare providers of the factors affecting maternal prenatal stress, making them aware of the probable vulnerabilities in the different regions in the country. Healthcare providers in Suriname also ought to consider integrating pre-natal perceived stress assessment as a routine part of prenatal care in primary care. Standard procedures for referral to specialized care are required for those pregnant women who screen as highly stressed. Most importantly, public health policy needs to address the regional factors associated with perceived stress to improve maternal and child health in Suriname.

## Supplementary Material

Supplementary Material Koendjbiharie et al Table S1: Comparison of participants with and without MacArthur social status scores (*n* = 1190)

## Figures and Tables

**Table 1.** The general characteristics of the CCREOH study population, stratified for geographic area (*n* = 1190).

Variables	Paramaribo	Interior	Nickerie	Total	*p*-value

	*n* = 738	*n* = 228	*n* = 224	*n* = 1190	
Cohen’s Perceived Stress Scores					

Median (IQR)	17 [13–20]	16 [9–20]	15 [12–18]	16 [12–20]	<0.001
	
20–40 high	29.8%	28.6%	18.8%	27.5%	0.006
0–19 low-normal	70.2%	71.4%	81.2%	72.5%	

Ethnic background					

Creole	32.2%	2.2%	7.2%	21.7%	
Hindustani	17.9%	0.0%	46.6%	19.9%	
Indigenous	1.5%	45.4%	6.3%	10.9%	< 0.001
Javanese	5.5%	0.4%	18.6%	7.0%	
Tribal people	20.3%	47.6%	0.5%	21.8%	
Mixed	22.6%	4.4%	20.8%	18.8%	

Age					

Mean ± SD	28.6 ± 6.3	27.0 ± 7.2	27.0 ± 5.7	28.0 ± 6.4	<0.001
	
16–19	9.9%	22.4%	12.1%	12.7%	<0.001
20–24	21.1%	21.5%	30.4%	22.9%	
25–29	28.3%	20.2%	23.2%	25.8%	
30–34	22.2%	21.5%	26.8%	22.9%	
35+	18.4%	14.5%	7.6%	15.6%	

Parity					

0–3 previous live births	90.4%	72.0%	93.7%	87.5%	<0.001
4+ previous live births	9.6%	28.0%	6.3%	12.5%	

Educational level					

Primary or not	11.8%	74.9%	9.0%	23.4%	<0.001
Lower secondary/vocational	36.7%	22.0%	36.7%	33.9%	
Upper secondary/vocational	31.2%	2.6%	42.5%	27.8%	
Tertiary	20.3%	0.4%	11.8%	14.9%	

Household income in SRD					

<800	6.6%	53.2%	7.3%	15.8%	<0.001
800–1499	16.6%	28.0%	21.8%	19.8%	
1500–2999	37.6%	11.0%	37.3%	32.4%	
3000+	39.2%	7.8%	33.6%	32.0%	

Household size					

<3 persons	12.9%	4.4%	13.1%	11.3%	0.001
3+ persons	87.1%	95.6%	86.9%	88.7%	

Marital status					

Married/living together	82.0%	96.0%	95.5%	87.2%	<0.001
Unmarried/single	18.0%	4.0%	4.5%	12.8%	

MacArthur social status score					

Mean ± SD	51.0 ± 18.1	33.8 ± 14.5	53.9 ± 15.7	48.0 ± 18.5	<0.001
	
0–30 low	9.9%	45.2%	5.2%	16.4%	<0.001
31–100 normal	90.1%	54.8%	94.8%	83.6%	

Associations were tested with the Chi-square test.

**Table 2.** Risk ratios (RR) with 95% confidence intervals (CI) for high perceived stress (*n* = 1190).

Predictors	High Perceived Stress				Total	RR	95% CI		Significance
	Yes		No				LB	UB	(α = 0.05)

	*n*	%	*n*	%					
Geographic area									

Paramaribo	218	29.8%	514	70.2%	732	1.58	1.18	2.13	0.006
Interior	64	28.6%	160	71.4%	224	1.52	1.08	2.14	
Nickerie	41	18.8%	177	81.2%	218	1			

Ethnic background									

Creole and Tribal	162	31.7%	349	68.3%	511	1.31	1.09	1.58	0.004
Else	159	24.1%	500	75.9%	659	1			

Age									

mean ± SD	27.3 ± 6.6		28.3 ± 6.3						0.015

16–19	55	37.9%	90	62.1%	145	1.46	1.15	1.84	0.003
20+	268	26.0%	761	74.0%	1029	1			

Parity									

0–3 previous live births	268	26.3%	751	73.7%	1019	1			0.021
4+ previous live births	52	35.6%	94	64.4%	146	1.35	1.06	1.72	

Educational level									

Lower (not/primary/lower secondary)	218	32.5%	452	67.5%	670	1.58	1.29	1.94	< 0.001
Higher (upper secondary/tertiary)	103	20.6%	397	79.4%	500	1			

Household income									

< 3000	226	29.6%	538	70.4%	764	1.36	1.09	1.70	0.006
3000+	78	21.7%	281	78.3%	359	1			

Household size									

<3 persons	38	28.6%	95	71.4%	133	1.05	0.79	1.40	0.453
3+ persons	282	27.3%	752	72.7%	1034	1			

Marital status									

Married/living together	270	26.5%	750	73.5%	1020	1			*0.062*
Unmarried/single	50	33.8%	98	66.2%	148	*1.28*	*1.00*	*1.63*	

MacArthur social status score									

mean ± SD	47.2 ± 20.9		48.3 ± 17.6						0.400

0–30 low	54	33.3%	108	66.7%	162	1.33	1.04	1.70	0.030
31–100 normal	208	25.1%	620	74.9%	828	1			

Associations were tested with the Chi-square test.

**Table 3.** Crude and adjusted odds ratios (OR) for high perceived stress with 95% confidence intervals (CI).

Crude Model	*p*-Value	Crude OR	95% CI	
			LB	UB
Geographic area	0.007			
	
Paramaribo vs. Nickerie	0.002	1.82	1.25	2.65
Interior vs. Nickerie	0.018	1.72	1.10	2.68
Adjusted model 1	*p*-Value	Adjusted OR	95% CI	
			LB	UB

Geographic area	0.030			
Paramaribo vs. Nickerie	0.008	1.72	1.15	2.57
Interior vs. Nickerie	0.042	1.62	1.02	2.59
Ethnic background (Creole and Tribal vs. else)	0.072	1.28	0.98	1.69
Adjusted model 2	*p*-Value	Adjusted OR	95% CI	
			LB	UB

Geographic area	0.020			
Paramaribo vs. Nickerie	0.007	1.75	1.17	2.62
Interior vs. Nickerie	0.162	1.41	0.87	2.27
Ethnic background (Creole and Tribal vs. else)	0.117	1.25	0.95	1.65
Maternal age (<20 vs. 20+)	0.001	1.92	1.31	2.80
Parity (4+ vs. 0–3 previous live births)	0.014	1.64	1.11	2.42
Adjusted model 3	*p*-Value	Adjusted OR	95% CI	
			LB	UB

Geographic area	0.007			
Paramaribo vs. Nickerie	0.011	1.70	1.13	2.57
Interior vs. Nickerie	0.741	1.09	0.66	1.78
Ethnic background (Creole and Tribal vs. else)	0.074	1.30	0.98	1.73
Maternal age (<20 vs. 20+)	0.055	1.48	0.99	2.22
Parity (4+ vs. 0–3 previous live births)	0.113	1.40	0.92	2.12
Education (lower vs. higher)	0.002	1.68	1.22	2.32
Income (<3000 vs. 3000+)	0.190	1.25	0.90	1.73
Adjusted model 4	*p*-Value	Adjusted OR	95% CI	
			LB	UB

Geographic area	0.005			
Paramaribo vs. Nickerie	0.022	1.68	1.08	2.62
Interior vs. Nickerie	0.750	0.91	0.53	1.59
Ethnic background (Creole and Tribal vs. else)	0.125	1.28	0.93	1.76
Maternal age (<20 vs. 20+)	0.046	1.55	1.01	2.38
Parity (4+ vs. 0–3 previous live births)	0.093	1.48	0.94	2.34
Education (lower vs. higher)	0.004	1.71	1.19	2.47
Income (<3000 vs. 3000+)	0.491	1.14	0.79	1.65
Social status score (low vs. normal)	0.156	1.36	0.89	2.06
Adjusted model 5 (final)	*p*-Value	Adjusted OR	95% CI	
			LB	UB

Geographic area	0.001			
Paramaribo vs. Nickerie	0.001	1.94	1.32	2.86
Interior vs. Nickerie	0.284	1.29	0.81	2.07
Maternal age (<20 vs. 20+)	0.019	1.57	1.08	2.28
Education (lower vs. higher)	<0.001	1.90	1.41	2.55

The final model included predictors that were significantly associated with the outcome variable based on the 95% confidence interval and *p*-value.

## Data Availability

The data that support the findings of this study are available upon reasonable request from the corresponding author.
