# Supplementary material for: Sociodemographic Influences on Perceived Stress during Pregnancy: Results from the CCREOH Environmental Epidemiologic Study, Suriname
Source: Women (Basel). Author manuscript; Available in PMC 2022 Sep 7. (PMC9451138; doi:10.3390/women2020014)
Supplement: Supplementary Material Koendjbiharie et al — Table S1: Comparison of participants with and without MacArthur social status scores (n = 1190) [file NIHMS1811106-supplement-Supplementary_Material_Koendjbiharie_et_al.pdf]

**Table S1. Comparison of participants with and without MacArthur social status scores (n=1190)**

| | MacArthur social status score | | | | |
|---|---|---|---|---|---|
| | **valid** | | **missing** | | |
| | n=990 | | n=200 | | |
| **Geographic area** | | | | | |
| Paramaribo | 588 | 59.4% | 150 | 75.0% | **<0.001** |
| Interior | 208 | 21.0% | 20 | 10.0% | |
| Nickerie | 194 | 19.6% | 30 | 15.0% | |
| **Cohen's Perceived Stress Scores** | | | | | |
| Mean ± SD | 15.8 ± 5.4 | | 16.9 ± 4.2 | | **0.009** |
| 20-40 high | 262 | 26.5% | 61 | 33.2% | *0.062* |
| 0–19 low–normal | 728 | 73.5% | 123 | 66.8% | |
| **Ethnic background** | | | | | |
| Creole and Tribal | 423 | 42.7% | 90 | 47.9% | *0.192* |
| Other ethnicities | 567 | 57.3% | 98 | 52.1% | |
| **Age** | | | | | |
| Mean ± SD | 27.9 ± 6.5 | | 28.3 ± 6.2 | | 0.458 |
| 16-19 | 131 | 13.2% | 20 | 10.0% | 0.210 |
| 20+ | 859 | 86.8% | 180 | 90.0% | |
| **Parity** | | | | | |
| 0–3 previous live births | 861 | 87.3% | 165 | 88.2% | 0.730 |
| 4+ previous live births | 125 | 12.7% | 22 | 11.8% | |
| **Educational level** | | | | | |
| Less educated | 578 | 58.4% | 97 | 51.6% | *0.085* |
| More educated | 412 | 41.6% | 91 | 48.4% | |
| **Household income in SRD** | | | | | |
| <3000 | 661 | 69.7% | 107 | 58.8% | **0.004** |
| 3000+ | 287 | 30.3% | 75 | 41.2% | |
| **Household size** | | | | | |
| <3 persons | 110 | 11.1% | 23 | 12.3% | 0.644 |
| 3+ persons | 878 | 88.9% | 164 | 87.7% | |
| **Marital status** | | | | | |
| Married/living together | 865 | 87.6% | 161 | 85.6% | 0.471 |
| Unmarried/single | 123 | 12.4% | 27 | 14.4% | |
